# Ulceration of the oral mucosa induced by antidepressant medication: a case report

**DOI:** 10.1186/1752-1947-3-98

**Published:** 2009-11-03

**Authors:** Fernanda Bertini, Nívea Cristina Sena Costa, Adriana Aigotti Haberbeck Brandão, Ana Sueli Rodrigues Cavalcante, Janete Dias Almeida

**Affiliations:** 1 Postgraduate Program in Biopathology and Department of Biosciences and Oral Diagnosis, São José dos Campos Dental School, São José dos Campos, São Paulo, Brazil; 2 Discipline of General Pathology, Department of Biosciences and Oral Diagnosis, São José dos Campos Dental School, São José dos Campos, São Paulo, Brazil; 3 Discipline of Stomatology, Department of Biosciences and Oral Diagnosis, São José dos Campos Dental School, São José dos Campos, São Paulo, Brazil

## Abstract

**Introduction:**

Ulcers are frequent lesions of the oral mucosa. Generally, they are circumscribed round or elliptical lesions surrounded by an erythematous halo and covered with an inflammatory exudate in their central portion, and are accompanied by painful symptoms. Oral ulcers affect 20% of the population, especially adolescents and young adults. The etiopathogenesis includes immunological alterations, infections, nutritional deficiency, trauma, food and contact allergies, autoimmune diseases, neoplasms, and psychosomatic, genetic and environmental factors.

**Case presentation:**

A 78-year-old Caucasian woman was referred by her dentist to our outpatient clinic with a 4-week history of an oral ulceration after using an antidepressant (sertraline hydrochloride). On the basis of the clinical findings and anamnesis, the occurrence of the lesion was attributed to the use of the drug. Exfoliative cytology was performed, to reassure the patient that it was not oral cancer, which revealed the presence of a nonspecific inflammatory reaction. The drug was replaced and resolution of symptoms was observed.

**Conclusion:**

Exfoliative cytology should be the complementary examination of choice in cases of oral ulcers with a suspicion of drug interaction. Although this is a rare event in dental practice, dentists should be aware of the diagnostic possibility of drug-induced ulcers and should cooperate with the clinician to adjust the prescribed medication to resolve the symptoms.

## Introduction

Oral ulcers are inflammatory lesions of the oral mucosa that affect approximately 20% of the population [1]. These ulcers are accompanied by painful symptoms and are generally characterized by shallow oval circumscribed lesions surrounded by an erythematous halo and covered with a fibrinous exudate in their central portion. Oral ulcers manifest as acute (duration of up to 6 weeks), chronic, or recurrent solitary or multiple lesions [2].

Numerous causes and factors involved in the formation of these lesions have been reported in the literature, including immunological alterations, infections, nutritional deficiency, repetitive trauma to the mucosa, food and contact allergies, autoimmune diseases and neoplasms, as well as psychosomatic, genetic and environmental factors [3].

Sertraline hydrochloride is an antidepressant agent used in clinical practice, which acts on the serotonin neurotransmitter. Antidepressants such as selective and potent serotonin reuptake inhibitors potentiate serotonergic neurotransmission and show a low frequency of adverse reactions [4].

A variety of cases of ulcerated lesions have been reported in the oral mucosa associated with the use of different systemic medications. This is of great interest to dentists who are frequently responsible for the diagnosis of these lesions. We report the case of a patient who developed oral ulceration after the use of an antidepressant (sertraline hydrochloride).

## Case presentation

**Figure 1:**
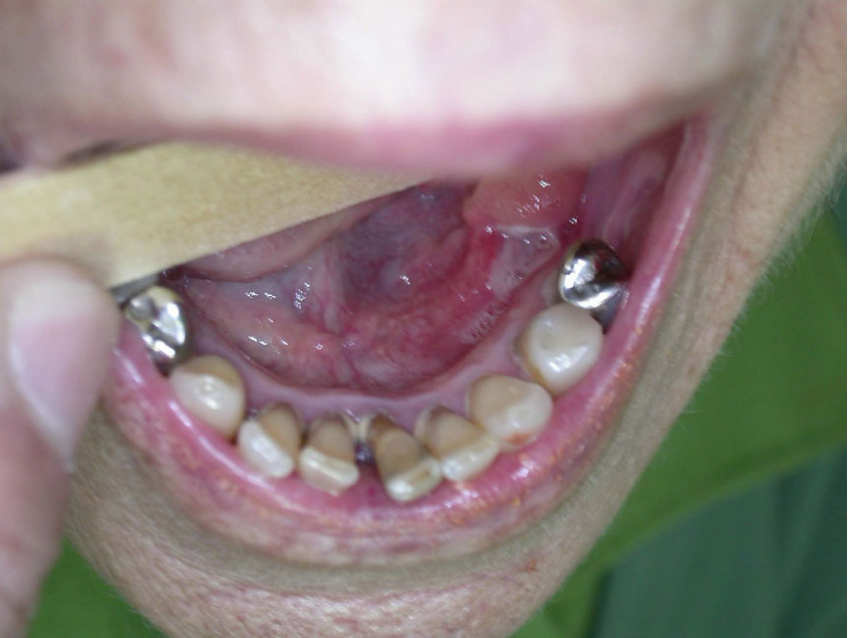
**Shallow ulcer with an erythematous border, measuring about 1.5 cm at its maximum diameter, located in the mucosa of the lingual region of the left lower premolars**.

A 78-year-old Caucasian woman was referred by her dentist to our outpatient clinic, with a 4-week history of an oral ulceration. She mentioned that she had basocellular carcinoma in her face and stomach cancer. Extra-oral clinical examination showed facial symmetry and palpable, mobile, smooth and asymptomatic submandibular lymph nodes. A shallow ulcer with an erythematous border, measuring about 1.5 cm at its maximum diameter, was noted upon intraoral examination. The ulcer was located in the mucosa of the lingual region of the left lower premolars (Figure 1) and was accompanied by painful symptoms.

Upon anamnesis, the patient reported the use of sertraline hydrochloride for the treatment of depression at an initial dose of 50 mg, which was subsequently increased to 100 mg when the occurrence of the lesion was first noted. After evaluation of the medication by the physician responsible, sertraline was replaced by 75 mg of venlafaxine and exfoliative cytology of the ulcer was performed.

**Figure 2:**
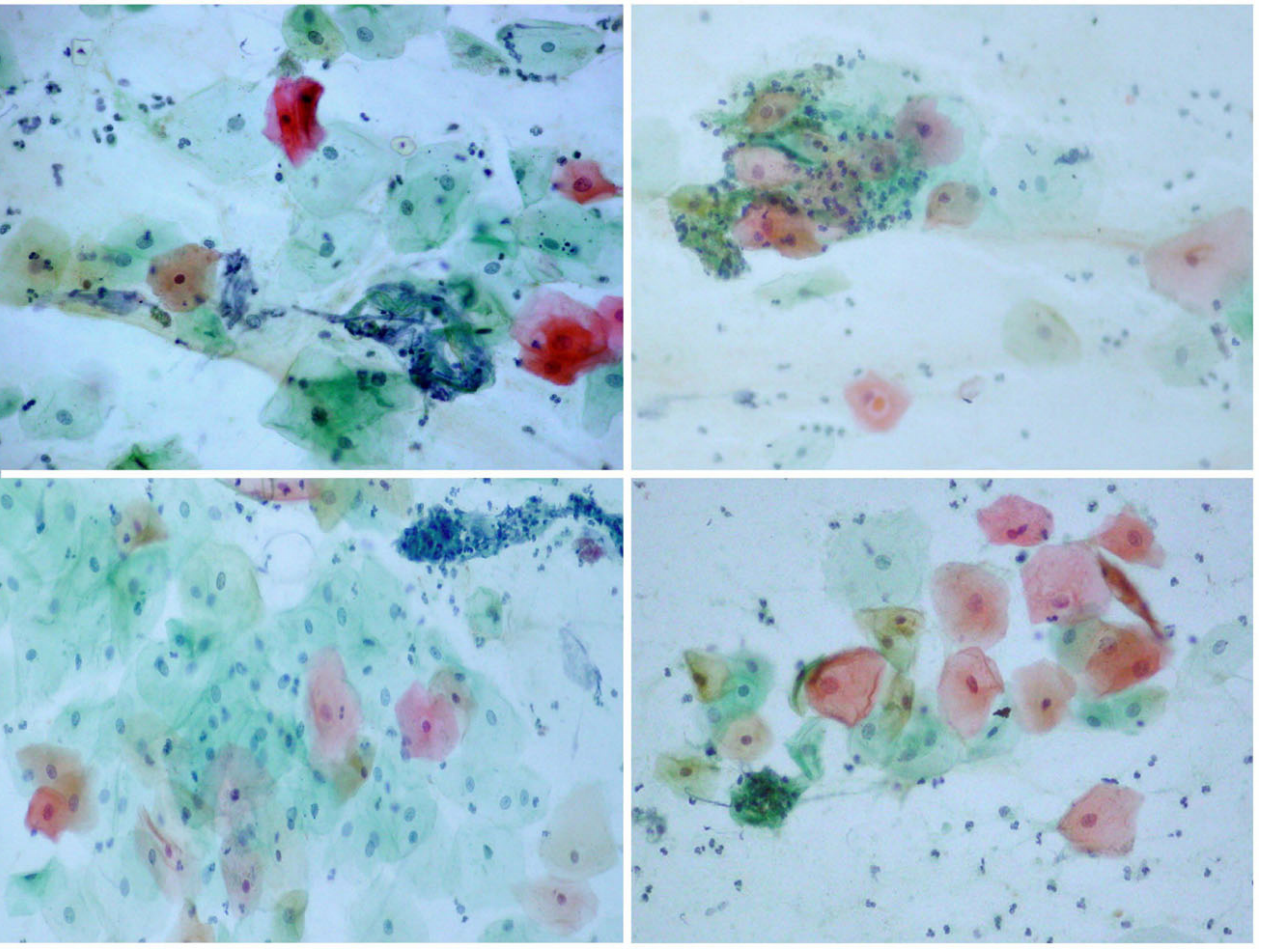
**Smears showing superficial and intermediate epithelial cells with inflammatory and degenerative alterations such as a perinuclear halo, cytoplasmic lysis and vacuolization, and numerous leukocytes, sometimes aggregated with filamentous mucus (Papanicolaou stain, 630×)**.

Exfoliative cytology of the lesion showed superficial and intermediate pavement epithelial cells presenting diverse inflammatory and degenerative alterations, such as vacuolization, a perinuclear halo, an enlarged nucleus, binucleation and lysis, numerous mono- and polymorphonuclear leukocytes, thick and filamentous mucus, and cell remnants, in addition to a mixed flora consisting of bacteria and *Candida *hyphae (Figure 2).

Mouth rinsing with a betamethasone elixir three times per day for 3 minutes was initially prescribed for a period of 5 days. On her return visit, the patient presented with improvement of the clinical symptoms and was instructed to continue mouth rinsing for an additional 7 days. Two weeks later, she returned, complaining of persistent pain in the affected area. The patient returned after 21 days showing significant improvement of the clinical symptoms and re-epithelization of the ulcer. The patient was followed up for 4 months at weekly intervals and was discharged after this period. She presented with no further oral complaints over 2 years of follow-up.

## Discussion

Our patient presented with an oral ulcer and was referred by her dentist, who was worried about the possibility of oral cancer. Oral ulcers can be the first manifestation of systemic diseases of immunogenetic origin, such as Behçet's disease [5] and others. Diseases such as pemphigus and pemphigoid may also impair differential diagnosis with nonspecific secondary ulcers after rupture of the bullae [6]. In such cases, a biopsy combined with immunofluorescence is a fundamental tool for a definitive diagnosis.

Deficiencies in iron, folic acid, B_12 _complex and B_6 _vitamins, and trace elements such as zinc have been variably related to the occurrence of ulcers [7].

Several studies have evaluated the use of drugs that may induce the formation of ulcers, including medications such as niflumic acid, captopril, piroxicam and phenobarbital. Cytotoxic agents used in antineoplastic therapies affect dividing cells, a phenomenon that manifests in the oral mucosa, inducing ulcers in some patients [8]. Alendronate, a drug prescribed for the treatment of patients with osteoporosis, has also been associated with the occurrence of oral ulcers. In a review of 200 patients investigating the adverse effects of drugs in the oral cavity, Smith and Burtner observed dry mouth in 80.5%, dysgeusia in 47.5% and stomatitis in 33.9% [9]. In another review on the adverse effects of drugs, Scully and Bagan reported adverse manifestations such as hypersalivation, white lesions, burning mouth sensation, mucositis, neoplasms, pemphigus, pemphigoid and other bullous disorders, mucosal pigmentation, lichenoid reactions, cheilitis, neuropathies, and halitosis [10]. The authors reported that aphthous ulcerations were observed after the use of β-blockers such as labetalol, captopril, nicorandil and nonsteroidal anti-inflammatory drugs and also after the use of mycophenolate or sirolimus, sodium lauryl sulfate, protease inhibitors, and sulfonamides.

In the era of transplantation, a frequent medical concern is the development of ulcers that might be exacerbated by the administration of immunosuppressive medications such as mycophenolate mofetil, which has been used in combination with calcineurin inhibitors and steroids. The drug-induced ulcerated lesions disappear when the medication is discontinued [11].

One study has shown the development of oral ulcers in four patients with angina pectoris who used nicorandil, a nicotinamide ester. In the cases studied, the lesions improved after dose reduction or interruption of the medication [12].

Stress has been identified as an important factor triggering the occurrence of oral ulcers, with the use of antidepressants being recommended in some patients for the control of recurrent aphthous ulcers [13]. However, in this study, after the clinical diagnosis of depression and prescription of sertraline hydrochloride, the patient developed an adverse reaction to the drug characterized by the occurrence of an ulcer in the oral mucosa. Replacement of the medication was requested, which led to the improvement of symptoms.

As the patient had a previous history of stomach cancer, she was upset about the possibility of the ulcer being an oral cancer lesion. Exfoliative cytology was performed to lessen the patient's worries about the lesion. Exfoliative cytology is a viable alternative and should be the complementary examination of choice in these situations. Because an early diagnosis of oral cancer is essential for survival and to minimize public health expenses, exfoliative cytology might be used as a complementary examination in the monitoring of cancer risk factors [14].

Oral cancer mainly affects the floor of the mouth, the lateral border of the tongue and the soft palate, although other areas of the mouth may also be involved. Many cases of oral cancer are diagnosed during the advanced phase, a fact that results in an unfavorable prognosis and high mortality, in addition to high costs of treatment and an increased number of complications. Thus, an early diagnosis and preventive approach are of extreme importance in this disease [15].

The clinical manifestations of adverse reactions to drugs depend on the dose and type of medication, as well as on individual differences related to the patient. These reactions might be rapid or persist for a number of days after the use of the drug. According to the literature and to clinical practice, in most drug-induced reactions improvement of clinical symptoms occurs after dose reduction or interruption of the medication. Generally, these adverse reactions occur in the first or second week after the beginning of the therapy of choice and depend on the dose and cumulative toxicity of the drug, with the reactions usually ranging from moderate to severe. However, severe reactions require rapid withdrawal of the drug or its replacement. Many patients use multiple systemic medications that may eventually be supplemented with other drugs necessary for dental treatment [10].

Oral ulcers are frequent in oral diagnosis clinics and the lesions must be carefully examined, including the aspect of the surface, the presence of an erythematous halo and the deepness of the lesion. The etiological diagnosis is based on the presence of associated signs and symptoms that should be investigated during anamnesis.

The dentist should have pharmacological knowledge of the prescribed drug and of its possible interaction with other medications. Thus, cooperation between the treating doctor and the dentist is necessary in order to choose the best treatment that will guarantee the well-being and best quality of life of the patient.

## Conclusion

Exfoliative cytology should be the complementary examination of choice in cases of oral ulcers with a suspicion of drug interaction. Although this is a rare event in dental practice, the dentist should be aware of the diagnostic possibility of drug-induced ulcers and should cooperate with the clinician to adjust the prescribed medication for symptom resolution.

## Consent

Written informed consent was obtained from the patient for publication of this case report and any accompanying images. A copy of the written consent is available for review by the Editor-in-Chief of this journal.

## Competing interests

The authors declare that they have no competing interests.

## Authors' contributions

JDA and FB analyzed and interpreted the patient data regarding the clinical aspects. AAHB performed the cytological examination of the smears, NCSC and ASRC were contributors in writing the manuscript. All authors read and approved the final manuscript.
